# Water retting process with hemp pre-treatment: effect on the enzymatic activities and microbial populations dynamic

**DOI:** 10.1007/s00253-024-13300-5

**Published:** 2024-09-13

**Authors:** Valeria Ventorino, Fatima Ezzahra Chouyia, Ida Romano, Mauro Mori, Olimpia Pepe

**Affiliations:** 1 Department of Agricultural Sciences, Division of Microbiology, University of Naples Federico II, Naples, Italy; 2 Force on Microbiome Studies, University of Naples Federico II, Naples, Italy; 3 Department of Agricultural Sciences, Division of Plant Biology and Crop Science, University of Naples Federico II, Naples, Italy

**Keywords:** *Cannabis sativa* L., Microbiota, Pectinolytics, Pectate lyase, Bacteria, Fungi

## Abstract

**Abstract:**

Proper retting process of hemp stems, in which efficient separation of cellulose fiber from the rest of the stem is promoted by indigenous microorganisms able to degrade pectin, is essential for fiber production and quality. This research aimed to investigate the effect of a pre-treatment dew retting in field of hemp stalks on the pectinolytic enzymatic activity and microbiota dynamic during lab-scale water retting process. A strong increase in the pectinase activity as well as in the aerobic and anaerobic pectinolytic concentration was observed from 14 to 21 days, especially using hemp stalks that were not subjected to a pre-retting treatment on field (WRF0 0.690 ± 0.05 U/mL). Results revealed that the microbial diversity significantly varied over time during the water retting and the development of microbiota characterizing the water retting of hemp stalks of different biosystems used in this study was affected by pre-treatment conditions in the field and water retting process and by an interaction between the two methods. Although at the beginning of the experiment a high biodiversity was recorded in all biosystems, the water retting led to a selection of microbial populations in function of the time of pre-treatment in field, especially in bacterial populations. The use of hemp stems did not subject to a field pre-treatment seems to help the development of a homogeneous and specific pectinolytic microbiota with a higher enzymatic activity in respect to samples exposed to uncontrolled environmental conditions for 10, 20, or 30 days before the water retting process.

**Key points:**

*• Microbial diversity significantly varied over time during water retting.*

*• Water retting microbiota was affected by dew pre-treatment in the field.*

*• Retting of no pretreated hemp allows the development of specific microbiota with high enzymatic activity.*

**Supplementary Information:**

The online version contains supplementary material available at 10.1007/s00253-024-13300-5.

## Introduction

Hemp (*Cannabis sativa* L.) is one of the oldest crops cultivated, with hemp tissues found more than 6000 years ago (Small 2015). Hemp is currently experiencing a renaissance due to its definition as multi-purpose crop; it is known for diverse array of phytochemicals, fibers, and agricultural characteristics, such as good resistance to pests and drought, a well-developed root system that prevents soil erosion, and a lower water requirement compared to other crops, such as cotton (Andre et al. 2016; Ely et al. 2022). It can play a role in satisfying the growing demand for biodegradable, durable fiber-based products due to the versatility of its fibers which can be used in textiles, yarns, paper, construction materials, auto parts, and composites (Johnson 2018; Shahzad 2012). Indeed, the fiber bundles in the phloem are one of the important hemp layers which support the conducting cells and give the stalk its sturdiness. In the bundles, the lignocellulosic fibers are found throughout the entire length of the stalk parallel to the vertical axis and integrated into a pectic polysaccharidic network (Crônier et al. 2005).

To obtain these fibers, they must be separated from the rest of the stem by a defibration operation known as retting process in which occurs the degradation of those substances (e.g., hemicellulose, pectin, and lignin) that bind the fiber containing tissues to the other components of the stalk as well as the fibers to each other (Angulu and Gusovius 2024; Ramesh 2018). This degradation can occur in a variety of ways, including dew retting in natural or controlled environment, water retting, osmotic degumming, enzymatic retting, steam explosion, and mechanical decortication (Lucas et al. 2022; Zimniewska 2022).

In the past, hemp stems were retted in rivers, but this practice was gradually outlawed and banned in several countries for water pollution concerns (Arufe et al. 2021). To overcome this problem and minimize environmental pollution, in Europe (mainly France) and North America, hemp is retted directly on the field (Angulu and Gusovius 2024; Arufe et al. 2021). Nowadays, water and field retting are the most common methods used to obtain high-quality fiber production and characterized by their low cost. For water retting, stems are removed from the field after harvesting and immersed in tank water, while for field retting, hemp stems are placed on the soil surface after harvest and are left to partially decompose (Lucas et al. 2022; Ramesh 2018). Both retting methods are carried out by the biological activity of indigenous microflora able to secrete several enzymes for the degradation of those substances that bind the fiber. The degradation of pectin, by a class of enzymes known as pectinases (i.e., polygalacturonases, pectic lyases, rhamnogalacturonases, and xylogalacturonases), is the key factor in the separation of fibers from the rest of the stem components since most of the fibers are surrounded by pectin-rich middle lamella and parenchyma cells (Angulu and Gusovius 2024). However, during retting, other various enzymes are also involved in the degradation process of hemp fiber components. The degradation of lignin, facilitated by enzymes such as laccases and peroxidases, is crucial for separating fiber bundles into individual fibers (Angulu and Gusovius 2024). Conversely, the degradation of cellulose by enzymes like endoglucanases, exoglucanases, and β-glucosidases is undesirable, as it can negatively impact the fiber characteristics that determine quality for many applications (Angulu and Gusovius 2024). Another important constituent to consider is hemicellulose, which is degraded by enzymes such as endo-β-1,4-xylanases, β-xylosidases, mannanases, and α-L-arabinofuranosidases. Distinguishing between the degradation of hemicelluloses and pectins in fiber plant tissues is challenging because the matrix embedding the microfibrillar cellulose scaffold consists of both pectins and non-cellulosic polysaccharides known as hemicelluloses (Angulu and Gusovius 2024). Both components are degraded early in the retting process, and this degradation is desirable (Arufe et al. 2021). Pectic enzymes secreted by indigenous microflora known as pectinolytic microorganisms, in which mostly bacteria and filamentous fungi developed in field retting are responsible for retting process (Angulu and Gusovius 2024; Tamburini et al. 2003). During the early stages of dew retting of hemp, fungi are considered the main players varying in species abundance as the process progressed, when bacteria outcompeted and dominated the process (Fernando et al. 2019; Liu et al. 2017) highlighting that both fungi and bacteria significantly contribute to the degradation of the matrix material, whereas, in water retting, the process seems to be driven by bacteria since no literature was found to report the role of fungi in contributing the degradation of the matrix material during water retting (Angulu and Gusovius 2024; Tamburini et al. 2003).

The retting time is an important key to obtaining high fiber quality, increasing the mechanical performance of fibers by avoiding cellulose degradation by microorganisms. The bacterial pectinolytic population present on hemp stems can develop during retting time by using the pectin richly present in hemp, carrying out the separation of the cellulose fibers from the shives. Moreover, combining the two maceration methods, considering a pre-retting in the field followed by retting in water, could accelerate the development of pectinolytic microbial populations and, therefore, the detachment of the cellulose fibers. Taking this into account, field pre-retting hemp stems were used to perform a lab-scale water retting process aimed to evaluate the development of aerobic and anaerobic bacterial pectinolytic populations, the dynamic of microbiota, and enzymatic activities during controlled process.

## Materials and methods

### Retting experiments

Hemp (*Cannabis sativa* L.) varieties Felina 32, Carmaleonte, and Futura 75 were grown at D’Amore farming in Frignano (Caserta, Campania, Italy; 41.015150 N, 14.175754 E). Hemp was planted on 29 April 2020 and harvested on 20 July 2020 receiving two supplemental irrigations. Hemp stem samples for the study were randomly selected.

**Fig. 1:**
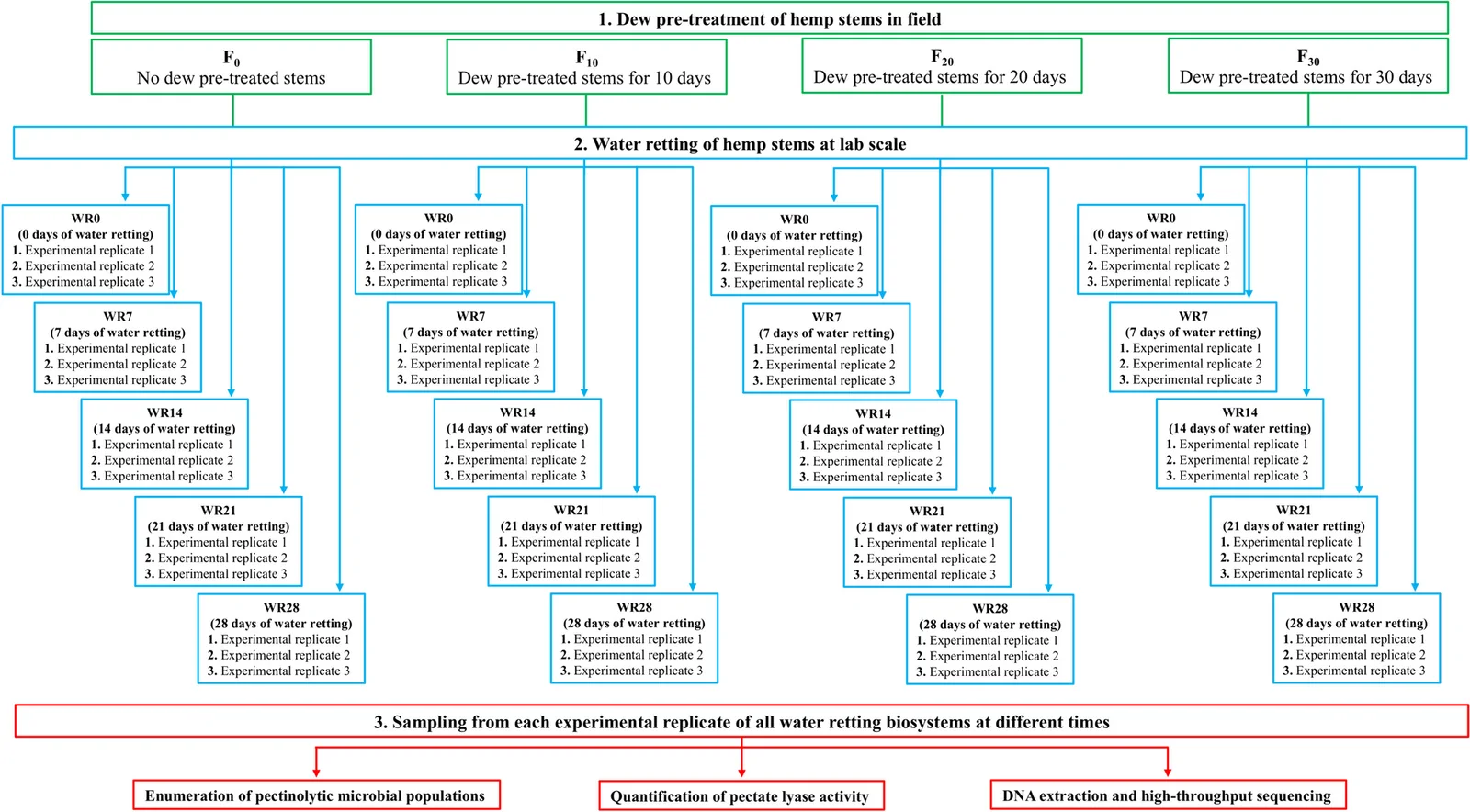
Scheme of experimental design and sampling procedures

Water retting experiments were conducted using 12-cm middle portion of the stems of the three hemp varieties (Felina 32, Carmaleonte and Futura 75) previously subjected to a dew pre-retting in field for 10 days (F10), 20 days (F20), or 30 days (F30). Hemp stems not subject to field retting treatment (F0) were also used as controls. A summary of experimental design and sampling procedures is reported in the Fig. 1. Hemp stems (three for each variety) were immersed in plastic tanks (length 15 cm, width 7 cm, and depth 10 cm) filled with 300 mL of the liquid medium containing 5 g/L pectin from citrus fruit (Sigma-Aldrich, Burlington, United States), 1 g/L NH_4_NO_3_, 1 g/L yeast extract, 50 mL/L standard salt solution (composition for 1 L: 5 g K_2_HPO_4_; 2.5 g MgSO_4_; 2.5 g NaCl; 0.05 g Fe_2_(SO_4_)_3_; and 0.05 g MnSO_4_), and 1 mL/L trace elements solution (composition for 1 L: 0.05 g K_2_MoO_4_·5H_2_O; 0.05 g Na_2_B_4_O_7_·10H_2_O; 0.05 g Co(NO_3_)_2_·6H_2_O; 0.05 g MnSO_4_; 0.05 g CdSO_4_; 0.05 g ZnSO_4_·7H_2_O; 0.05 g CuSO_4_·H_2_O; and 0.1 g FeCl_3_) (Giacobbe et al. 2014). All water retting trials were conducted in triplicate and incubated at 30 °C for 28 days. Samples were withdrawn at the beginning the water retting process (WR0) and after 7 (WR7), 14 (WR14), 21 (WR21), and 28 (WR28) days and used for cultural and molecular microbiological analyses as well as enzymatic assays.

### Enumeration of pectinolytic microbial populations

Microbiological counts were performed on serially diluted samples which were spread on the surface of a solid medium containing pectin as sole carbon source (Ventorino et al. 2018a). Plates were incubated for 7 days at 30 °C under aerobic or anaerobic conditions (Oxoid’s Anaerogen™ System, Oxoid, Basingstoke, UK). After incubation, plates were flooded with 1% of hexadecyltrimethylammonium bromide solution for 20–30 min. Pectinolytic were detected by the development of a clear zone around the colonies.

### Assessment of pectinolytic activity during water retting process

From each sample, 1.5 mL of retting liquid substrate was recovered and centrifuged at 5000 × *g* for 5 min at 4° C, and 500 μL of the supernatant was used to measure pectinase activity. Enzymatic activity was determined by Pectate lyase kit (Megazyme Ltd, Bray, Ireland) using polygalacturonic acid (Megazyme Ltd, Bray, Ireland) as substrate, following the supplier’s instructions. The activity was monitored spectrophotometrically by measuring the increase in adsorption at 300 nm of the reaction mixture by referring to a standard curve. The analytical determinations correspond to the mean value of three replicates.

### DNA extraction and high-throughput sequencing

Total genomic DNA was extracted from hemp stem samples at the beginning (WR0) and at 7 (WR7), 14 (WR14), 21 (WR21), and 28 (WR28) days of water retting process using stems previously subjected to a dew pre-retting in field for 10 days (F10), 20 days (F20), or 30 days (F30) and did not subject to field retting treatment (F0). Three independent experimental replicates for each condition and time, for a total of 60 samples, were used for DNA extraction as summarized in the Fig. 1. The Fast DNA SPIN Kit for Soil (MP Biomedicals, Illkirch Cedex, France) was used according to the manufacturer’s instructions. The microbial diversity was evaluated by amplicon-based metagenomic sequencing using the primers S-D-Bact-0341F50 (5′-CCTACGGGNGGCWGCAG-3′) and S-D-Bact-0785R50 (5′-GACTACHVGGGTATCTAATCC-3′) (Klindworth et al. 2013) for the V3-V4 region of the 16S rRNA gene and the primers EMP.ITS1 (5′- CTTGGTCATTTAGAGGAAGTAA-3′) and EMP.ITS2 (5′-GCTGCGTTCTTCATCGATGC-3′) (Bokulich and Mills 2013) for the ITS1-5.8S-ITS2 fungal region. PCR conditions for bacteria and fungi were performed as previously described (Gugliucci et al. 2023). PCR products were purified with the AgencourtAMPure beads (Beckman Coulter, Milan, IT) and quantified using an AF2200 Plate Reader (Eppendorf, Milan, IT). Equimolar pools were obtained, and sequencing was carried out on an Illumina MiSeq platform, yielding to 2 × 250 bp, paired end reads.

### Bioinformatics and data analysis

After sequencing, the raw reads were imported and analyzed in QIIME 2 software (Bolyen et al. 2019). Sequence adapters and primers were trimmed by using cut adapter, whereas the DADA2 algorithm (Callahan et al. 2016) was used to trim low quality reads, to remove chimeric sequences, and joined sequences shorter than 250 bp by using the DADA2 denoise paired plugin of QIIME2. Amplicon sequence variants (ASVs) obtained by DADA2 were rarefied at the lowest number sequences/sample and used for taxonomic assignment using the QIIME feature-classifier plugin against Greengenes and UNITE database for bacteria and fungi, respectively. Chloroplast and mitochondria contaminants and singleton ASVs were removed, and relative abundances of the other taxa were recalculated.

Alpha diversity was assessed with Shannon diversity. The Shannon–Weaver index (*H*) is calculated as follows: *H* =  − sum *pi* * ln *pi*, where *pi* is the proportional abundance of species *i*. Additionally, from the Shannon–Weaver index, the diversity was calculated as follows: *D* = exp(*H*) (Jost 2007; Bodenhausen et al. 2013). ANOVA (*P* ≤ 0.05) was used to assess the difference in the Shannon–Weaver index. Beta diversity was examined by permutational multivariate analysis of variance (PERMANOVA) using the adonis function from vegan. Principal coordinate analysis (PCoA) on Bray–Curtis dissimilarities was used to visualize the differences between samples.

Bar-plots were generated using R packages phyloseq 1.46.0 (McMurdie and Holmes 2013) and ggplot2 3.5.1 (Wickham 2016). Furthermore, the metabolic function was predicted by Tax4Fun analysis through Kyoto Encyclopedia of Genes and Genomes (KEGG) database (Wang et al. 2019). Analysis mainly focused on the differences of predicted abundances of genes involved in pectin degradation during the water retting process of samples no or subjected to the field pre-treatment. ANOVA (*P* ≤ 0.05) was used to assess the difference in predicted abundance taking into account the factors field pre pre-retting time and water pre-retting time. Heatmaps were generated in R using the package Pheatmap 1.0.12 (Kolde 2019).

The raw data have been deposited in the Sequence Read Archive Database of the National Center of Biotechnology Information (PRJNA1124099).

### Statistics

To assess differences in the pectinolytic microbial enumeration and enzymatic activity among samples, a one-way ANOVA by Tukey’s HSD post hoc for pairwise comparison of means (at* P* < 0.05) was performed using the SPSS 21.0 software package (SPSS Inc., Cary, NC, USA).

## Results

### Microbial enumeration of pectinolytic populations

The results obtained at the beginning of lab-scale water retting trial (0 days) allowed to have information about the effect of the pre-retting field treatment on the concentration of aerobic and anaerobic pectinolytic populations. Using 10 and 20 days of field pre-retting hemp stalks, the highest values in the range of 6.10–6.52 log CFU (colony-forming unit)/mL were observed (Table 1) in the water process. However, in general, aerobic pectinolytics predominated over anaerobic population from 7 to 28 days of water retting process. They constantly increased during lab-scale water retting process reaching values up to about 10^7^ CFU/mL at 14 days of maceration that remained quite stable until the end of the process with all pre-retting hemp stalks (Table 1).

**Table 1:** Development of pectinolytic microorganisms throughout the water retting process of hemp stems with and without pre-retting treatment on fields

Time of water retting (days)	Viable count (Log CFU/mL)							
	WRF0		WRF10		WRF20		WRF30	
	Aerobic	Anaerobic	Aerobic	Anaerobic	Aerobic	Anaerobic	Aerobic	Anaerobic
**0**	5.09 ± 0.02^**h**^	5.09 ± 0.01^**h**^	6.10 ± 0.02^**g**^	6.52 ± 0.01^**c**^	6.30 ± 0.05^**fg**^	6.20 ± 0.00^**de**^	5.29 ± 0.01^**h**^	5.40 ± 0.00^**g**^
**7**	5.97 ± 0.01^**g**^	2.00 ± 0.00^**m**^	6.47 ± 0.02^**f**^	2.01 ± 0.01^**m**^	6.42 ± 0.00^**f**^	2.03 ± 0.01^**m**^	6.60 ± 0.00^**ef**^	2.02 ± 0.02^**m**^
**14**	6.78 ± 0.02^**e**^	3.18 ± 0.02^**l**^	7.16 ± 0.04^**d**^	5.49 ± 0.00^**g**^	7.22 ± 0.06^**cd**^	5.99 ± 0.04^**ef**^	7.40 ± 0.03^**bc**^	3.76 ± 0.01^**i**^
**21**	6.93 ± 0.00^**de**^	5.78 ± 0.01^**f**^	7.45 ± 0.03^**b**^	6.12 ± 0.02^**e**^	7.39 ± 0.05^**bc**^	6.21 ± 0.05^**de**^	7.52 ± 0.02^**b**^	6.12 ± 0.04^**e**^
**28**	7.29 ± 0.01**c**	6.36 ± 0.01^**cd**^	7.50 ± 0.04^**b**^	7.21 ± 0.00^**b**^	7.92 ± 0.06^**a**^	7.42 ± 0.01^**ab**^	7.69 ± 0.05^**ab**^	7.59 ± 0.03^**a**^

Different letters in aerobic or anaerobic populations indicate significant differences according to Tukey’s HSD (*P* < 0.05)

*WRF0* water retting hemp stems without pre-retting treatment on fields

*WRF10* water retting hemp stems with pre-retting treatment on fields for 10 days

*WRF20* water retting hemp stems with pre-retting treatment on fields for 20 days

*WRF30* water retting hemp stems with pre-retting treatment on fields for 30 days

The anaerobic pectinolytic population decreased of about 3–4 log after 7 days of water retting process reaching values of 10^2^ CFU/mL in all the samples, after that a progressive increase up 10^7^ CFU/mL was recorded at 28 days of maceration (Table 1).

### Pectate lyase activity

**Fig. 2:**
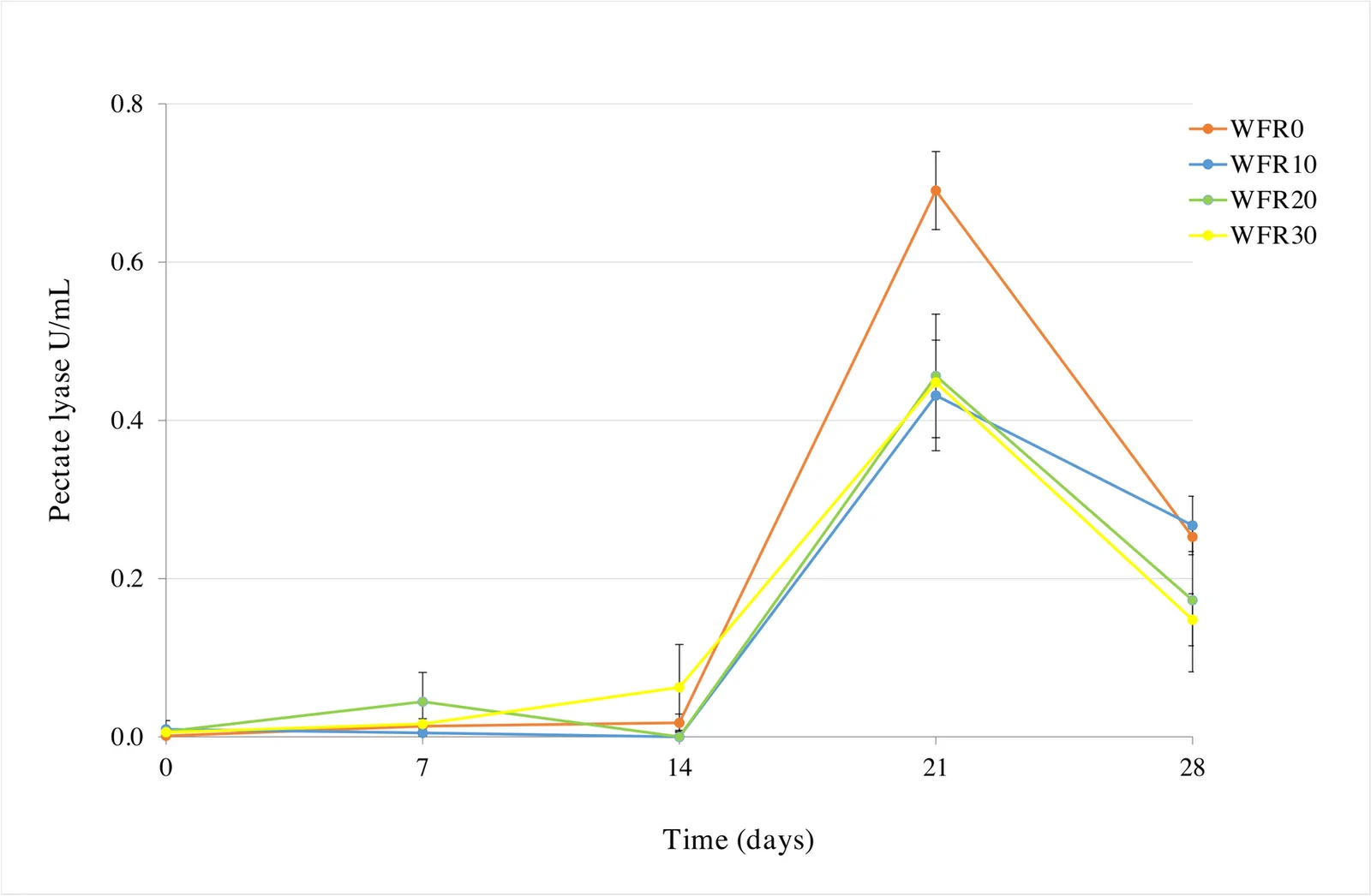
Pectate lyase activity during water hemp retting process. WFR0, water retting hemp stems without pre-retting treatment on fields; WFR10, hemp stems with pre-retting treatment on fields for 10 days; WFR20, hemp stems with pre-retting treatment on fields for 20 days; WFR30, hemp stems with pre-retting treatment on fields for 30 days

The pectinase activity was very low if not absent until 14 days of the water retting process, and no significant differences were recorded between pre-retting and no pre-retting samples (Fig. 2). A strong increase in the pectinase activity was observed from 14 to 21 days in all water retting conditions but above all using hemp stalks was not subjected to a pre-retting on field (WR_F0_ 0.690 ± 0.05 U/mL). The other samples showed a lower pectinase activity ranging from 0.432 ± 0.07 to 0.456 ± 0.08 U/mL (Fig. 2). From 21 to 28 days, a general significant decrease of pectinase activity up to about 0.20 U/mL was observed (Fig. 2).

### Microbial community diversity

**Fig. 3:**
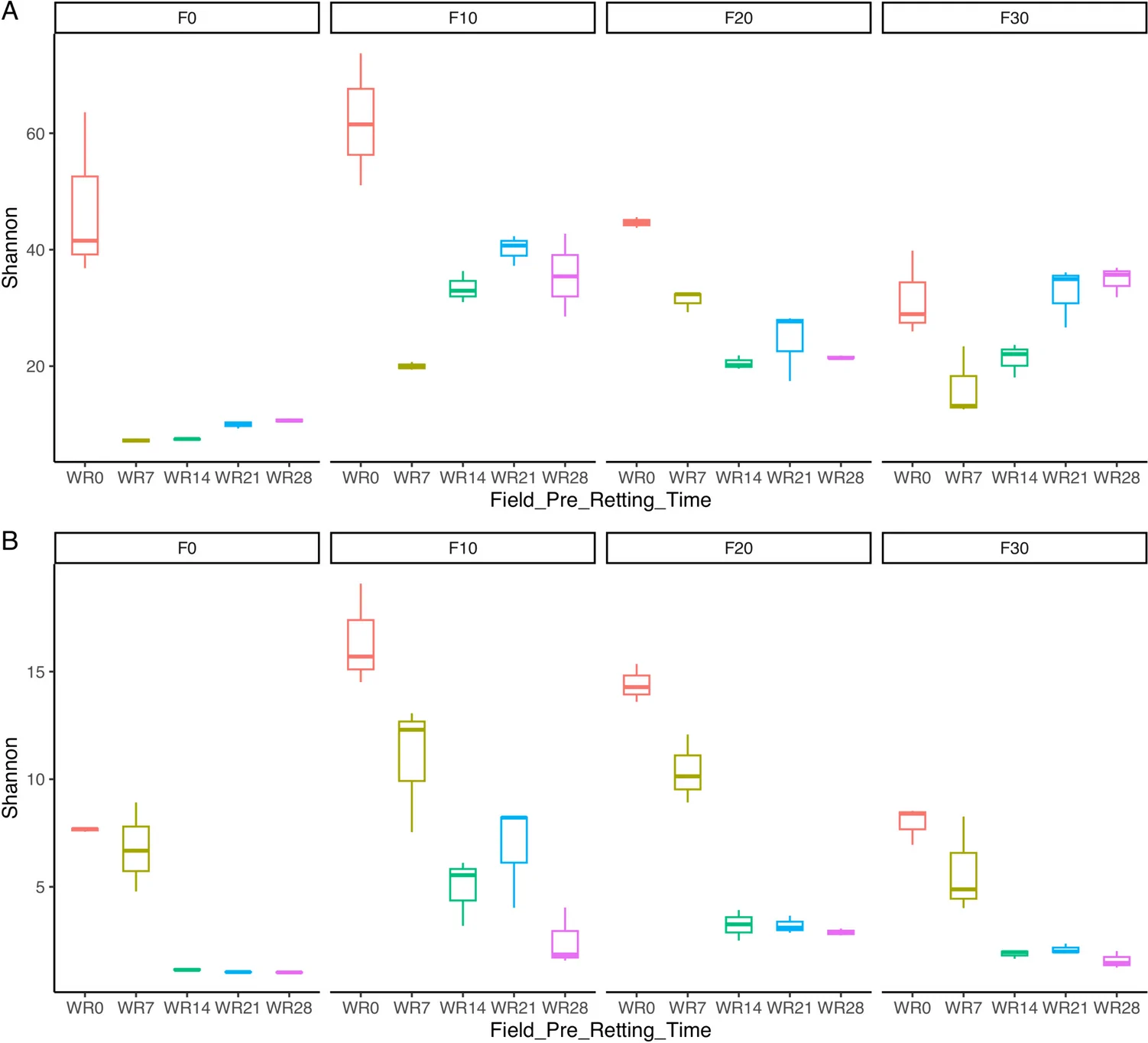
The box plots showing Shannon diversity index based on bacterial (**A**) and fungal (**B**) communities in the different biosystems during hemp retting process. F0, stems without pre-retting treatment on fields; F10, hemp stems with pre-retting treatment on fields for 10 days; F20, hemp stems with pre-retting treatment on fields for 20 days; F30, hemp stems with pre-retting treatment on fields for 30 days; WR0, water retting at the beginning of the process; WR7, water retting at 7 days of the process; WR14, water retting at 14 days of the process; WR21, water retting at 21 days of the process; WR28, water retting at 28 days of the process

The microbial diversity was characterized by partial 16S rRNA gene or 18S rRNA gene sequencing obtained from DNA extracted from the water hemp retting biosystems during the process. In total, 5,054,152 and 1,990,215 high quality reads were analyzed for bacteria and fungi, respectively. The alpha-diversity was determined by calculating the Shannon diversity index based on ASVs of 99% identity (Fig. 3). The statistical analysis (Supplemental Table S1) according to ANOVA indicated that both bacterial and fungal diversity were affected by pre-retting field treatment (*P* < 0.001) and water retting process (*P* < 0.001) as well as by an interaction between the two methods (*P* < 0.001). Results revealed that the bacterial diversity significantly varied over time during the water retting showing the highest Shannon diversity at the beginning of the process (W0) in the biosystems with samples no subjected to field pre-treatment (F0) and pre-treated for 10 and 20 days (F10 and F20) (*P* < 0.001; Fig. 3a). In the biosystems with the hemp treated in field for 30 days (F30) were observed less differences from day 0 to 28 of water retting (Fig. 3a). In addition, biosystems with no pre-retting field treated samples showed the lowest bacterial diversity from day 7 to 28 of water retting (*P* < 0.001; Fig. 3a). Fungal diversity exhibited a similar trend, exhibiting a significant decrease of biodiversity from 14 to 28 days as well as the lowest values in the biosystem with no pre-treated hemp (Fig. 3b).

**Fig. 4:**
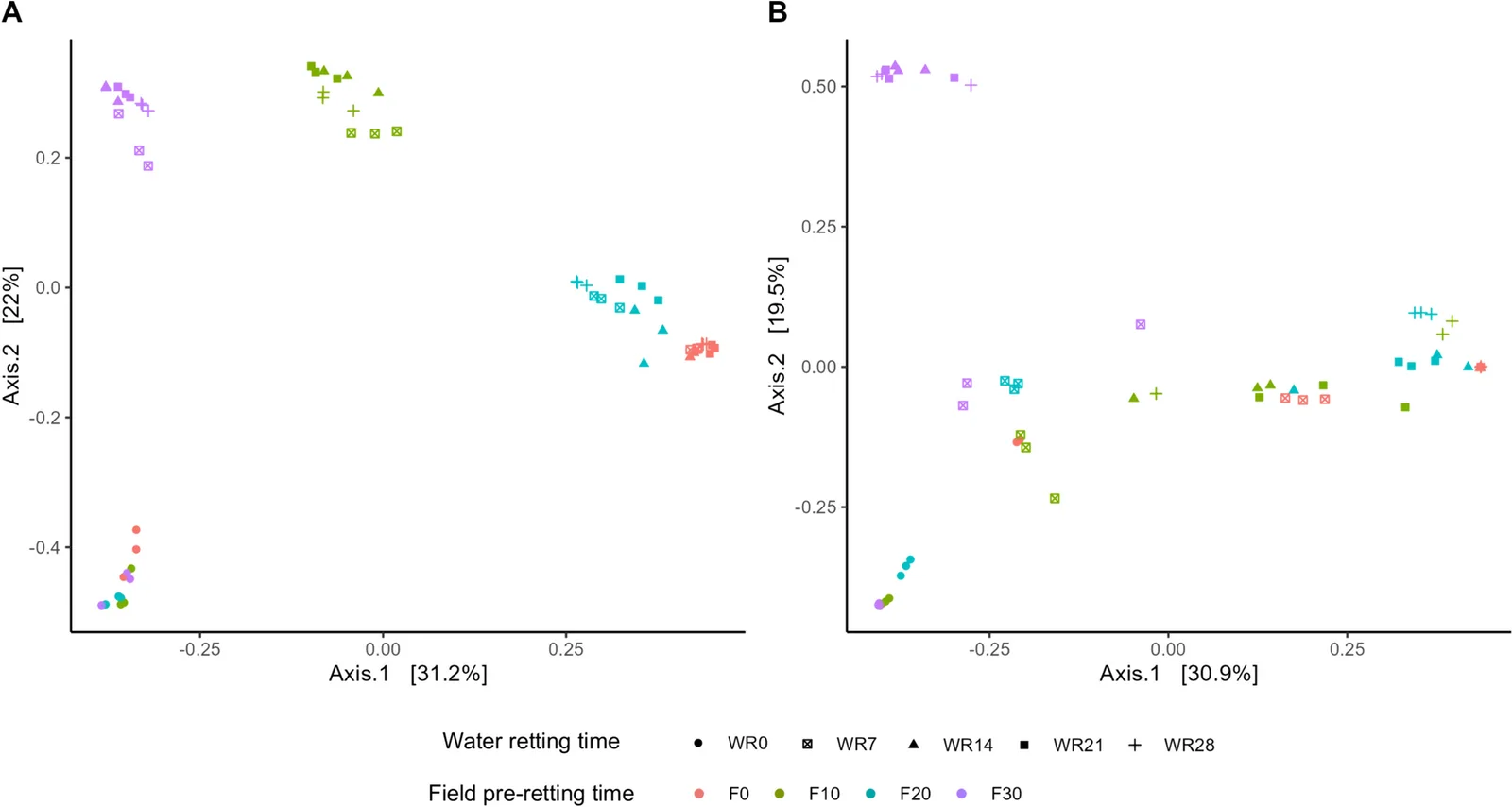
Principal Coordinates Analysis (PCoA) on Bray–Curtis dissimilarities of bacterial (**A**) and fungal (**B**) communities in the different biosystems during hemp retting process. F0, stems without pre-retting treatment on fields; F10, hemp stems with pre-retting treatment on fields for 10 days; F20, hemp stems with pre-retting treatment on fields for 20 days; F30, hemp stems with pre-retting treatment on fields for 30 days; WR0, water retting at the beginning of the process; WR7, water retting at 7 days of the process; WR14, water retting at 14 days of the process; WR21, water retting at 21 days of the process; WR28, water retting at 28 days of the process

Beta diversity was estimated on the basis of Bray–Curtis dissimilarities and highlighted a marked significant difference among the bacterial microbiota in the biosystems. As shown in the Fig. 4a, the PCoA of bacterial populations showed five principal groups based on interactions between the time of hemp pre-treatment in field (F0, F10, F20, and F30) and of water retting (WR0, WR7, WR14, WR21, and WR28). In fact, a significant difference of biodiversity (*P* < 0.001; Supplemental Table S2) was revealed for the samples at the beginning of the water retting process (W0) of all biosystems, regardless the pre-retting treatment in field, creating a separated group (Fig. 4a). The other four principal groups were based on the time of hemp pre-retting in field (F0, F10, F20, and F30; *P* < 0.001; Supplemental Table S2). Moreover, within each of these major group, sample distribution seems to be influenced by the water retting time (*P* < 0.001; Supplemental Table S2).

PCoA of fungal populations showed a similar trend in which was generated a cluster comprising the samples at the beginning of the water retting process (W0) of all biosystems (Fig. 4b). However, the distribution of the other samples was not marked as for bacteria, even if PERMANOVA analysis allowed to identify distinct fungal community for all experimental factors applied (Supplemental Table S2). Water retting time exerted a gradual influence on fungal diversity in the biosystems with the hemp subjected to 30 days of field pre-retting, exhibiting a distinct group that included the samples at 14, 21, and 28 days of water retting process (Fig. 4b).

### Microbial taxonomic composition

To evaluate any alteration in the microbial communities’ structure during water retting process of hemp samples subjected or not to the field pre-treatment, the relative abundances of bacterial and fungal taxa were determined at family and genus level.

#### Bacteria

**Fig. 5:**
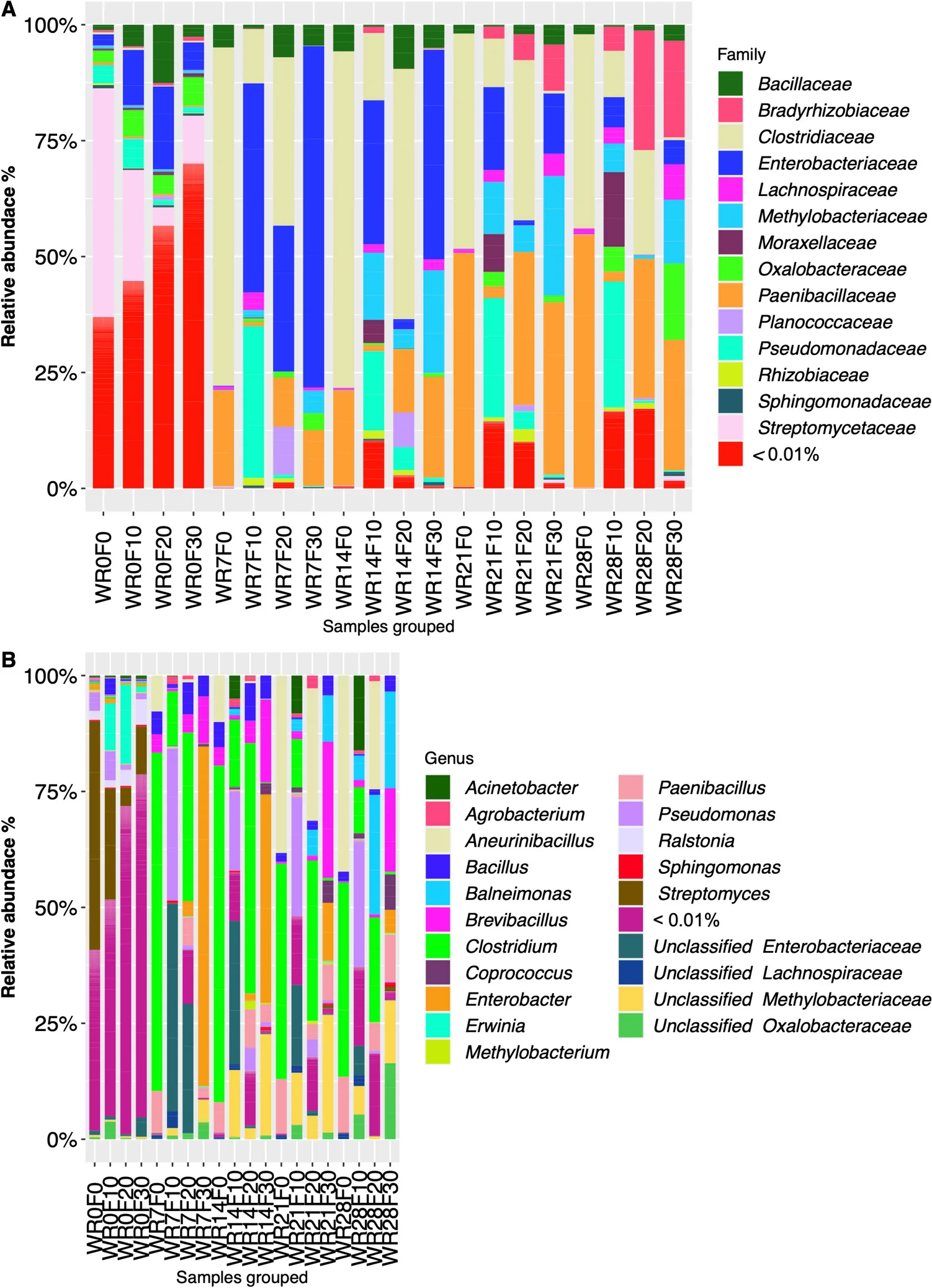
Relative abundance of bacterial families (**A**) and genera (**B**) in the different biosystems during hemp water retting process. WR0F0, beginning of the water retting process without pre-retting on field; WR7F0, water retting at 7 days without pre-retting treatment on field; WR14F0, water retting at 14 days without pre-retting on field; WR21F0, water retting at 21 days without pre-retting on field; WR28F0, water retting at 28 days without pre-retting on field; WR0F10, beginning of the water retting process with pre-treatment on field for 10 days; WR7F10, water retting at 7 days with pre-treatment on field for 10 days; WR14F10, water retting at 14 days with pre-treatment on field for 10 days; WR21F10, water retting at 21 days with pre-treatment on field for 10 days; WR28F10, water retting at 28 days with pre-treatment on field for 10 days; WR0F20, beginning of the water retting process with pre-treatment on field for 20 days; WR7F20, water retting at 7 days with pre-treatment on field for 20 days; WR14F20, water retting at 14 days with pre-treatment on field for 20 days; WR21F20, water retting at 21 days with pre-treatment on field for 20 days; WR28F20, water retting at 28 days with pre-treatment on field for 20 days; WR0F30, beginning of the water retting process with pre-treatment on field for 30 days; WR7F30, water retting at 7 days with pre-treatment on field for 30 days; WR14F30, water retting at 14 days with pre-treatment on field for 30 days; WR21F30, water retting at 21 days with pre-treatment on field for 30 days; WR28F30, water retting at 28 days with pre-treatment on field for 30 days

In total, 14 different bacterial families were detected in the samples with a relative abundance > 0.01% (Fig. 5a). The results of bacterial taxonomic analysis demonstrated that at the beginning of water retting process (WR0) the samples of all biosystems (WR0F0, WR0F10, WR0F20, and WR0F30) had the highest significantly biodiversity showing from about 35% to 65% of bacterial taxa with a relative abundance < 0.01%. Moreover, at this time, bacterial diversity was dominated by *Streptomycetaceae*, especially in the no pre-treated biosystem (WR0F0), accounting for about 49.32 ± 0.14% of the total biodiversity (Fig. 5a). After 7 days, the bacterial families that characterized the beginning of the process drastically decreased over time and biodiversity significantly reduced and changed according to field pre-treatment. No pre-treated biosystem showed the lowest biodiversity in which spore-forming families such as *Clostridiaceae* and *Paenibacillaceae* accounted for > 90% of the total biodiversity, followed by *Bacillaceae* (about 5%), remaining quite stable until the end of the process (WR28F0; Fig. 5a). Field pre-treated samples were characterized not only by *Clostridiaceae*, *Paenibacillaceae*, and *Bacillaceae*, but also by an increase of *Enterobacteriaceae* up to about 30–70% and 30–45% at 7 (WR7F10, WR7F20, and WR7F30) and 14 (WR14F10, WR14F20, and WR14F30) days of water retting process. Biosystems with samples subjected to a field pre-treatment for 10 (F10) and 30 (F30) days, after 14 days showed an increase of *Methylobacteriaceae* (about 15–20%), remaining quite stable until the end of the water retting process (Fig. 5a).

The microbial diversity was also analyzed at a deeper taxonomic level. In particular, the analysis of the sequences at genus level, allow to identify 16 bacterial genera and 5 unclassified taxa with a relative abundance > 0.01% (Fig. 5b). These taxa showed a different relative abundance depending on the water retting time and field pre-treatment. A dominance of *Streptomyces* in no pre-treated samples was observed at the beginning of the water retting process (WR0F0) accounting for 49.32 ± 0.14% % of the total diversity (Fig. 5b). After 7 days (WR7F0), an increase of the relative abundance of *Clostridium* was detected (about 72.96 ± 0.01%), decreasing at 21 (WR21F0) and 28 days (WR28F0) up to about 46.44 ± 0.01% and 41.88 ± 0.01%, respectively. Interestingly, a simultaneous constant increase of *Aneurinibacillus* (relative abundance of 38.27 ± 0.01% and 42.28 ± 0.03% in WR21F0 and WR28F0, respectively) and *Paenibacillus* (relative abundance of 11.65 ± 0.01% and 11.85 ± 0.01% in WR21F0 and WR28F0, respectively) was recorded (Fig. 5b). Bacterial microbiota of the pre-treated biosystems was characterized by a higher biodiversity and variability according to field pre-retting time. In detail, *Acinetobacter*, *Clostridium*, *Pseudomonas*, unclassified *Enterobacteriaceae*, and unclassified *Methylobacteriaceae* dominated the entire water retting process of samples pre-treated in field for 10 days (Fig. 5b). Samples subjected at a field pre-treatment for 20 days were characterized by dominance of *Bacillus, Brevibacillus, Clostridium*, *Paenibacillus*, and *Pseudomonas* genera during the entire water retting process. Finally, a significant increase of *Enterobacter* (up to about 73.27 ± 0.13%) at 7 days of water retting process in the biosystem with hemp stems pre-treated in field for 30 days (WR7F30) was observed and then a constant decrease up to at the end of the process (Fig. 5b).

#### Fungi

**Fig. 6:**
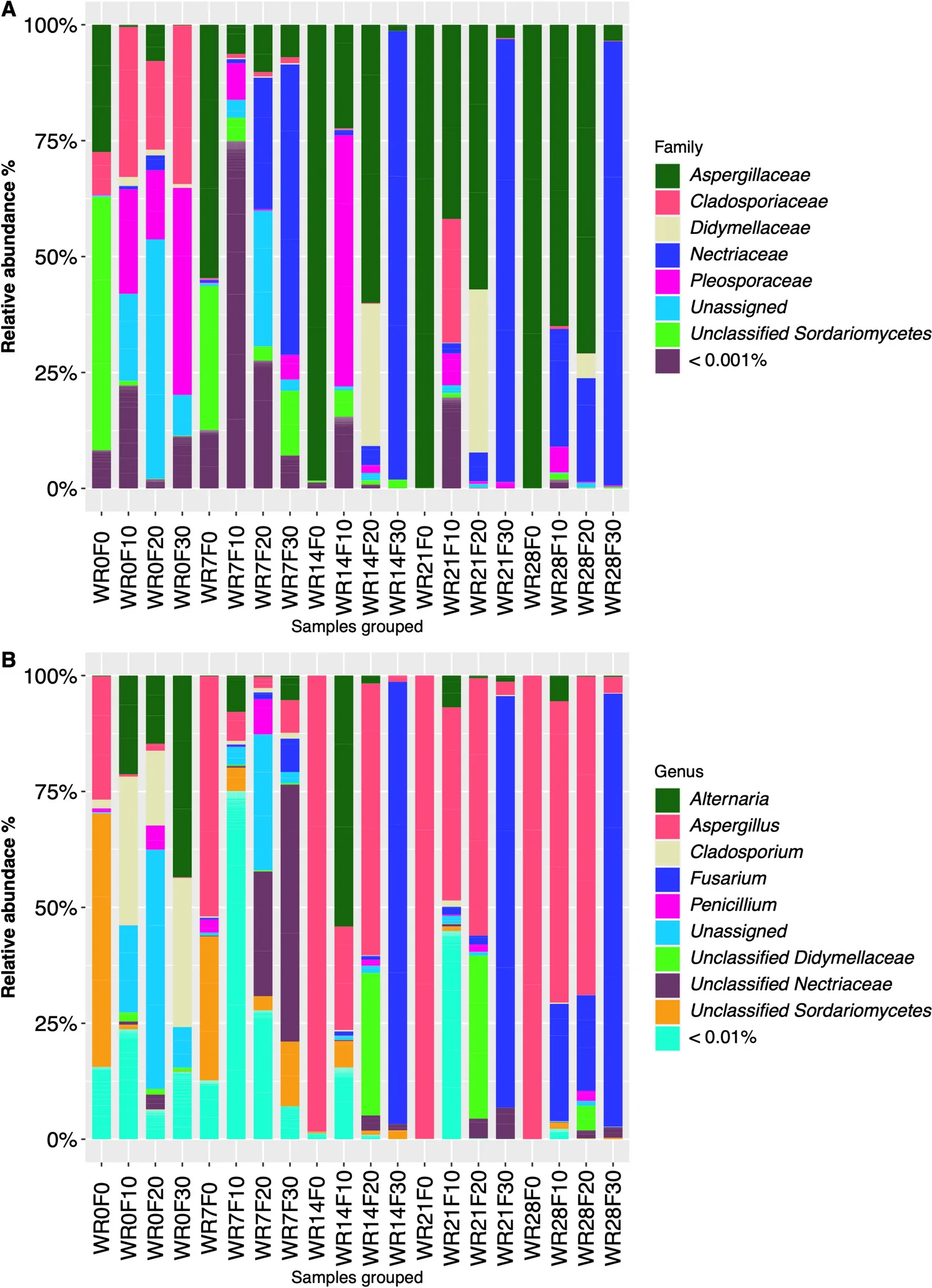
Relative abundance of fungal families (**A**) and genera (**B**) in the different biosystems during hemp retting process. WR0F0, beginning of the water retting process without pre-retting on field; WR7F0, water retting at 7 days without pre-retting treatment on field; WR14F0, water retting at 14 days without pre-retting on field; WR21F0, water retting at 21 days without pre-retting on field; WR28F0, water retting at 28 days without pre-retting on field; WR0F10, beginning of the water retting process with pre-treatment on field for 10 days; WR7F10, water retting at 7 days with pre-treatment on field for 10 days; WR14F10, water retting at 14 days with pre-treatment on field for 10 days; WR21F10, water retting at 21 days with pre-treatment on field for 10 days; WR28F10, water retting at 28 days with pre-treatment on field for 10 days; WR0F20, beginning of the water retting process with pre-treatment on field for 20 days; WR7F20, water retting at 7 days with pre-treatment on field for 20 days; WR14F20, water retting at 14 days with pre-treatment on field for 20 days; WR21F20, water retting at 21 days with pre-treatment on field for 20 days; WR28F20, water retting at 28 days with pre-treatment on field for 20 days; WR0F30, beginning of the water retting process with pre-treatment on field for 30 days; WR7F30, water retting at 7 days with pre-treatment on field for 30 days; WR14F30, water retting at 14 days with pre-treatment on field for 30 days; WR21F30, water retting at 21 days with pre-treatment on field for 30 days; WR28F30, water retting at 28 days with pre-treatment on field for 30 days

The family-level taxonomic analysis of the fungal community showed that the development of specific taxa was influenced by the interaction between water retting time and field pre-treatment (Fig. 6a). Specifically, unclassified *Sordariomycetes* (54.64 ± 0.01%) dominated the no pre-treated samples at the beginning of the process (WR0F0), followed by *Aspergillaceae* (27.45 ± 0.02%) and *Cladosporiaceae* (9.18 ± 0.01%). During water retting process, a constant increase of *Aspergillaceae* relative abundance was observed reaching values up to about 99% at the end of the experiment (WR28F0; Fig. 6a). The pre-treated biosystems, although at the beginning of the experiment (WR0F10, WR0F20, and W0F30) showed a similar taxonomic composition dominated by *Cladosporiaceae*, *Pleosporaceae*, and unassigned fungi, during the water retting process fungal populations differentially developed. *Aspergillaceae* and *Nectriaceae* accounted for about 90% of the total fungal diversity at the end of the water retting process of samples subjected to the field pre-treatment for 10 (WR28F10) and 20 (WR28F20); whereas the only *Nectriaceae* family (relative abundance of about 95%) dominated the water retting process of samples subjected to the field pre-treatment for 30 days (WR14F30, WR21F30, and WR28F30) (Fig. 6a).

By analyzing the fungal taxa at deeply taxonomic level, *Aspergillus* dominated the process of the no pre-treated samples achieving values of about 98.29 ± 0.01% after 14 days (Fig. 6b). Samples subjected to the field pre-treatment were characterized by unassigned or unclassified taxa at 7 and 14 days of water retting process (WR7F10, WR7F20, WR7F30, WR14F10, WR14F20, and WR14F30), after that *Aspergillus* and *Fusarium* dominated the end of the process of WR28F10 (relative abundance of 65.02 ± 0.38 and 25.30 ± 0.26%, respectively) and WR28F20 (relative abundance of 68.71 ± 0.05% and 20.63 ± 0.06%, respectively) samples, whereas *Fusarium* accounted for 93.42 ± 0.06% of the total fungal diversity in WR28F30 (Fig. 6b).

### Functional prediction analysis

**Fig. 7:**
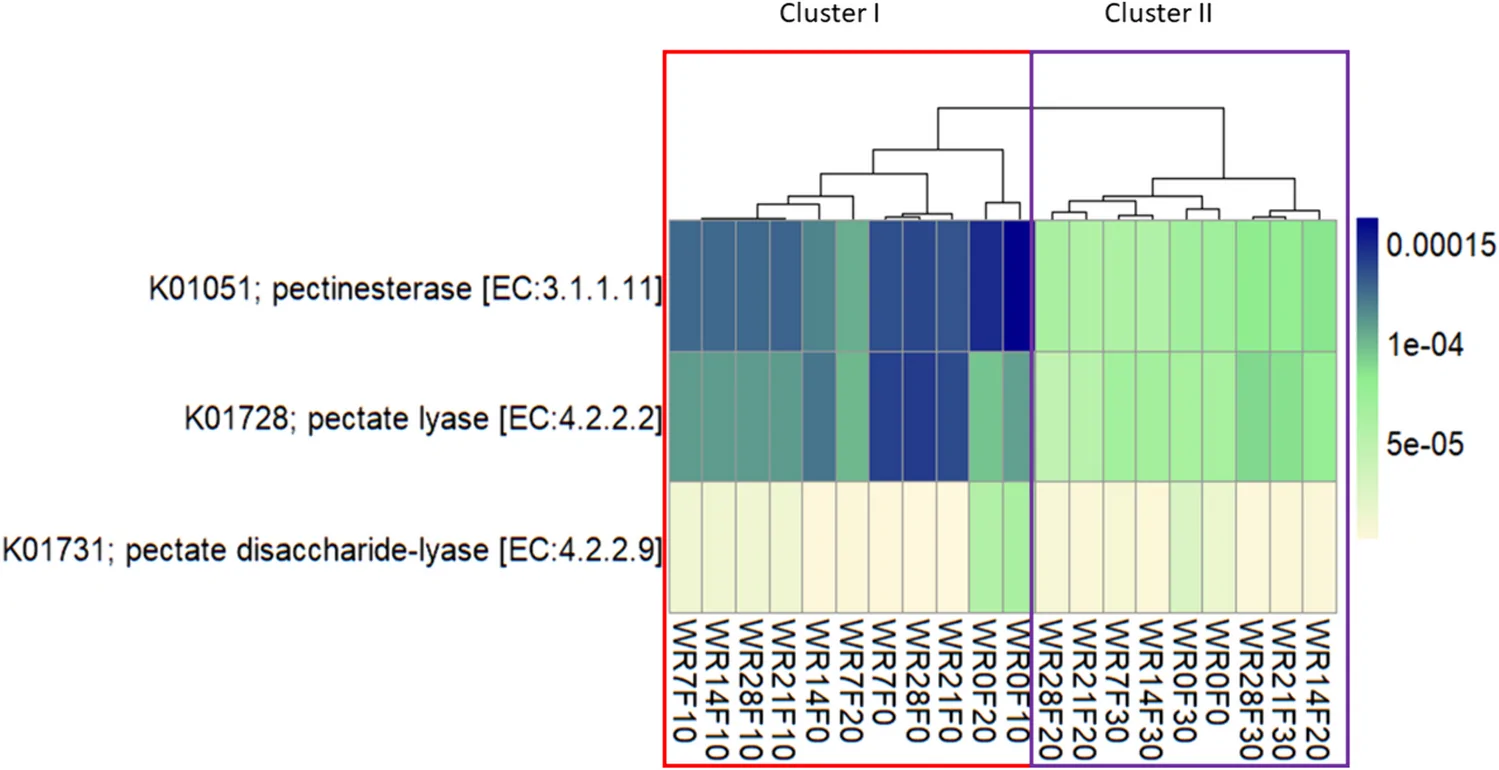
Predicted abundances of pectinesterase (EC:3.1.1.11), pectate lyase (EC:4.2.2.2), and pectate disaccharide-lyase (EC:4.2.2.9) genes. The color code refers to gene abundance, with high predicted abundances (blue) and low predicted abundances (light yellow). WR0F0, beginning of the water retting process without pre-retting on field; WR7F0, water retting at 7 days without pre-retting treatment on field; WR14F0, water retting at 14 days without pre-retting on field; WR21F0, water retting at 21 days without pre-retting on field; WR28F0, water retting at 28 days without pre-retting on field; WR0F10, beginning of the water retting process with pre-treatment on field for 10 days; WR7F10, water retting at 7 days with pre-treatment on field for 10 days; WR14F10, water retting at 14 days with pre-treatment on field for 10 days; WR21F10, water retting at 21 days with pre-treatment on field for 10 days; WR28F10, water retting at 28 days with pre-treatment on field for 10 days; WR0F20, beginning of the water retting process with pre-treatment on field for 20 days; WR7F20, water retting at 7 days with pre-treatment on field for 20 days; WR14F20, water retting at 14 days with pre-treatment on field for 20 days; WR21F20, water retting at 21 days with pre-treatment on field for 20 days; WR28F20, water retting at 28 days with pre-treatment on field for 20 days; WR0F30, beginning of the water retting process with pre-treatment on field for 30 days; WR7F30, water retting at 7 days with pre-treatment on field for 30 days; WR14F30, water retting at 14 days with pre-treatment on field for 30 days; WR21F30, water retting at 21 days with pre-treatment on field for 30 days; WR28F30, water retting at 28 days with pre-treatment on field for 30 days

Functional profiles were predicted based on the 16S rRNA gene sequencing data to assess differences between the different biosystems. Although this analysis allowed us to analyze over 6000 functional genes, predicted abundances of some interesting enzyme‐encoding genes associated with pectin degradation were reported (Fig. 7). In detail, analysis focused on three functional genes as pectinesterase (EC:3.1.1.11), pectate lyase (EC:4.2.2.2), and pectate disaccharide-lyase (EC:4.2.2.9).

Functional profiles of the microbial communities were clustered into two major groups clearly associated to the field pre-treatment (Fig. 7). Cluster 1 included water retting samples no subjected to field pre-treatment, and pre-treated for 10 and 20 days (WR7F0, WR14F0, WR21F0, WR28F0, WR0F10, WR7F10, WR14F10, WR21F10, WR28F10, WR0F20, and WR7F20) in which the predicted abundances of the genes for the pectinesterase (EC:3.1.1.11) and pectate lyase (EC:4.2.2.2) were highest, above all in the samples WR7F0, WR14F0, WR21F0, and WR28F0. The cluster 2 grouped the other samples subjected to field pre-treatment for 20 days and for 30 days (WR14F20, WR21F20, WR28F20, WR0F30, WR7F30, WR14F30, WR21F30, and WR28F30) in which the enzymatic activities analyzed were predicted with a lower level. However, the predicted abundance of pectate disaccharide-lyase (EC:4.2.2.9) was low in all the samples excepting for WR0F10 and WR0F20 (Fig. 7).

## Discussion

Recent ecological practices tend to use sustainable renewable and environmentally friendly materials, including the use of natural cellulosic fibers as an alternative to synthetic materials in composites. Retting process facilitates the extraction of hemp fibers from the central woody part of the stems by preserving their quality during mechanical decortications (Paridah et al. 2011; Sisti et al. 2018). Microorganisms play an important role in degrading pectin (degumming), which is abundantly present in the middle lamella, by producing a range of specific enzymes allowing progressive fiber separation (Akin et al. 2007). However, the retting process must be further researched to develop high-performance hemp bio-composites and to promote hemp fiber as sustainable alternative to cotton and petroleum-based synthetic fibers for textile raw material (Gedik and Avinc 2020). It is important to highlight the potential of sustainable hemp fiber production in the textile industry as 67 million tons of synthetic fibers and 26 million tons of cotton fibers were produced in 2018, with cotton production having significant environmental impacts due to the massive use of irrigation and pesticides (Ely et al. 2022). The development of the natural pectinolytic microbial populations present on the hemp stems with specific functional activities can control the retting process through the degradation of pectin of the plant cell wall releasing the hemp fiber. Several works have been conducted to investigate the role and the dynamic of indigenous microflora driving the retting process of fibrous plants by molecular approaches (Ribeiro et al. 2015; Chabbert et al. 2020; Djemiel et al. 2017, 2020; Law et al. 2020). A key factor for the significance of the results is the methodological aspect of nucleic acid extraction. Recently, Bou Orm et al. (2023) investigated the efficiency of three different protocols for DNA recovery and abundance of bacterial and fungal populations from hemp stems after dew retting in field highlighting that the commercial kit FastDNA™ Spin Kit for soil, also used in this work, was suitable for the evaluation of microbiota during the process. However, although recent studies on retting microbiology of several fibrous plants are developed, to the best of our knowledge this is the first work that investigate microbiota and enzymatic activity combining dew and water retting processes.

During the water retting process conducted at lab-scale, we observed that aerobic pectinolytic populations were higher in the biosystems containing hemp subjected to field pre-retting treatment (WFR10, WFR20, and WFR30). However, the highest pectinolytic enzyme activity was detected in the biosystems containing untreated hemp (WFR0). This result could be explained by the effect of dew retting process in the field, where it is challenging to control parameters such as temperature and air humidity. These uncontrolled conditions could alter the proper development of the pectinolytic population as dew retting is carried out not only by pectinolytic bacteria but also by fungal colonies (Angulu and Gusovius 2024). However, although aerobic pectinolytics significantly increased from 0 to 7 days, anerobic populations decreased and then progressively increased until the end of the process. This result could be due to the presence of oxygen at the beginning of the water retting process and therefore to the increase more in aerobic populations than anaerobic. As the retting process progressed, the consumption of oxygen in the broth favored the conditions of anaerobiosis, increasing anaerobic taxa. The consumption of oxygen in a short time favored the development of anaerobic spore-forming members belonging to the *Clostridiaceae* family (Tamburini et al. 2003) with an evident dominance during the water retting process of all biosystems analyzed in this study. However, in contrast to drew retting, the water retting process used in this study allowed us to develop and increase the natural aerobic and anaerobic pectinolytic populations which could be due to the consumption of pectin present in the hemp stems as well as in the broth as a carbon source. The results obtained are in according with Bacci et al. (2011) and Sanjay et al. (2019) who reported that the water retting is a widely practiced method that tend to well degrade the pectin and produce a better quality fiber while the drew retting process suffers from many drawbacks because of its dependence on the weather which risks to damage the fibers. Although water retting poses a risk of environmental pollution and it is banned in several countries, the development and optimization of retting processes under controlled conditions, such as using tanks or bioreactors implemented with back-slopping technique and/or water recycling, could significantly reduce environmental damage, offering a sustainable solution for large-scale applications. As previously stated pectinolytic microorganisms are involved in the degradation of pectin by producing a range of enzymes such as pectin methylesterase, pectinase, and pectin lyases, and they can convert the pectic substances into constituent monosaccharides or specific oligosaccharides without the production of undesirable products (Khattab 2022; Patidar et al. 2018) by accelerating the process. The pectate lyase is one of several inducible enzymes, mostly produced by microorganisms and known to degrade highly esterified pectin without the aid of additional pectic enzymes (Burns 1991). In this study, the pectinolytic activity was monitored by measuring the pectate lyase enzyme and demonstrated a similar trend between the samples by showing their highest activity from 14 to 28 days in the water sample without pre-treatment on field. The dynamics of pectate lyase activity revealed in this study is consistent with previous studies in which the enzymatic activities related to pectins and hemicelluloses measured during hemp retting under controlled conditions (Bleuze et al. 2018) or flax dew retting (Chabbert et al. 2020) increased especially during the first 14 days and then declined. This could be due to the presence of a recalcitrant pectin structure and/or lower accessibility due to the presence of a lignin-encrusted middle lamella component between the fibers (George et al. 2016). Results of enzyme activity was also confirmed by the functional prediction analysis in which the predicted abundances of the genes for the pectinesterase (EC:3.1.1.11) and pectate lyase (EC:4.2.2.2) were highest in the samples WR7F0, WR14F0, WR21F0, and WR28F0. The highest enzymatic activity recorded at 21 days could be due to the increase in the number of aerobic and anaerobic microorganisms capable of degrading the pectin. Moreover, a member of *Clostridiaceae* family, as *Clostridium*, is known to have a high pectinolytic activity. From water retting of hemp fibers, Tamburini et al. (2003) isolated a total of 24 anaerobic strains assigned to the *Clostridium* genus and all demonstrated a high pectinolytic activity. However, also members belong to the *Paenibacillaceae* family, known to have a marked pectinolytic activity in aerobic and anaerobic conditions, could contributed to high activity in the present study. In fact, some spore-forming genera belonging *Paenibacillaceae*, as *Paenibacillus*, exhibit a facultative aerobic or anaerobic metabolism (Giacobbe et al. 2014; Tamburini et al. 2003). The increase of pectate lyase activity could be also due to absolute dominance of members belong to the phylum *Ascomycota* (relative abundance from 90 to 99%), widely known as producer of pectinase enzyme (Campos-Rivero et al. 2019; Khattab 2022). In fact, fungal species can entry through damaged areas by hyphae and synthetizing extracellular cutinises are able to destroy the cuticular layer (Manian et al. 2021). The most common genus used in industrial production is *Aspergillus* with the species *Aspergillus niger* (Khattab 2022), *Aspergillus tubingensis* (Huang et al. 2019), and *Aspergillus flavus* (Anisa et al. 2013).

The development of microbiota characterizing the water retting of hemp fibers of different biosystems used in this study is affected by pre-treatment conditions in the field. In fact, although at the beginning of the experiment a high biodiversity was recorded in all biosystems, the water retting process led to a selection of microbial populations in function of the time of pre-treatment in field, especially in bacterial populations (Figs. 3 and 4). The different responses of bacterial and fungal populations could be due to the lower sensitivity of fungi to any environmental changes because they generally show longer generation times than bacteria and therefore respond more slowly to environmental perturbation (Ventorino et al. 2018b). In particular, at the beginning of the process no pre-treated samples were dominated by *Streptomycetaceae* (mainly *Streptomyces*) while *Proteobacteria* dominated primarily pre-treated biosystems. These results agree with previous metabarcoding investigation during hemp dew retting in which *Actinobacteria* was reported as the dominant phylum in unretted stem samples (Bou Horm et al. 2023), while *Proteobacteria* among dominant phyla of retted stems (Bou Horm et al. 2023; Law et al. 2020). However, during the water retting process an increase of *Bacilli* (mainly *Clostridiaceae*, *Paenibacillaceae*, and *Bacillaceae*) was observed in all the biosystems. Taxa associated to *Actinobacteria*, *Firmicutes*, and *Proteobacteria* phyla were also previously identified by Zhao et al. (2016) during flax water retting. Hou and Liu (2012) reported that the common dominant groups present during water retting were *Clostridiaceae*, *Pseudomonadaceae*, and *Bacillaceae*. However, in our study, an increase in taxa belonging to *Proteobacteria* phylum (especially *Gammaproteobacteria*) was also recorded in the biosystems with stems pre-retted in field. OTUs identified as belonging to *Proteobacteria* and *Actinobacteria* phyla were also identified in previous research during flax dew retting (Chabbert et al. 2020; Djemiel et al. 2017). Dominant phyla identified were *Proteobacteria*, *Bacteroidetes*, *Actinobacteria*, and *Firmicutes*, among bacterial populations, and *Ascomycota*, *Basidiomycota*, and *Zygomycota*, among fungal populations (Djemiel et al. 2017). In the present work, all biosystems were dominated by *Ascomycota* (mainly *Aspergillaceae*, *Cladosporiaceae* and *Nectriaceae* families). *Dothideomycetes* and *Sordariomycetes*, belonging to *Ascomycota* phylum, were reported as the major classes present during flax dew retting (Chabbert et al. 2020). Ribeiro et al. (2015) reported that the most frequent fungal sequences recovered during dew retting of hemp stems were principally related to the genera *Cladosporium*.

In conclusion, from 14 and 21 days of water retting process, it corresponds to the maximum pectinolytic population and pectinase activity to optimize the recovery of the fiber and to limit enzymatic degradative action on the cellulose. Moreover, the use of hemp fibers not subject to a pre-treatment in field seems to help the development of a homogeneous and specific pectinolytic microbiota with a higher enzymatic activity in respect to samples exposed to uncontrolled environmental conditions for 10, 20, or 30 days before the water retting process.

## Supplementary Information

Below is the link to the electronic supplementary material.

Supplementary file1 (PDF 89 KB)

## Data Availability

The raw data have been deposited in the Sequence Read Archive Database of the National Center of Biotechnology Information (PRJNA1124099).
