# Supplementary material for: Water retting process with hemp pre-treatment: effect on the enzymatic activities and microbial populations dynamic
Source: Appl Microbiol Biotechnol. 2024 Sep 13;108(1):464. doi: 10.1007/s00253-024-13300-5 (PMC11399178; doi:10.1007/s00253-024-13300-5)
Supplement: Supplementary file 1 — Supplementary file1 (PDF 89 KB) [file 253_2024_13300_MOESM1_ESM.pdf]

Supplemental material

Applied Microbiology and Biotechnology

Water retting process with hemp pre-treatment: effect on the enzymatic activities and microbial populations dynamic

Valeria Ventorino[1,2,§,*], Fatima Ezzahra Chouyia[1,§], Ida Romano[1], Mauro Mori[3], Olimpia Pepe[1,2]

[1] Department of Agricultural Sciences, Division of Microbiology, University of Naples Federico II, Naples, Italy

[2] Force on Microbiome Studies, University of Naples Federico II, Naples, Italy

[3] Department of Agricultural Sciences, Division of Plant Biology and Crop Science, University of Naples Federico II, Naples, Italy

* Corresponding author: Valeria Ventorino; e-mail: valeria.ventorino@unina.it; Phone: +39(0)812539404

[§]V Ventorino and FE Chouyia contributed equally to this work and share first authorship.

Table S1. Analysis of Variance of Shannon Index for bacteria (A), and fungi (B) in the different biosystems during hemp retting process.

**A) Bacteria**	**df**	**Sum Sq**	**Mean Sq**	**F value**	**Pr(>F)**	
Field retting time	3	3548.5	1182.85	41.2392	2.587e-12	***
Water retting time	4	5823.1	1455.77	50.7545	3.772e-15	***
Field retting time : Water retting time	12	2537.3	211.44	7.3718	6.747e-07	***
**B) Fungi**	**df**	**Sum Sq**	**Mean Sq**	**F value**	**Pr(>F)**	
Field retting time	3	244.49	81.496	41.6448	2.233e-12	***
Water retting time	4	846.23	211.558	108.1068	< 2.2e-16	***
Field retting time : Water retting time	12	84.93	7.077	3.6166	0.001061	**
Signif. codes: 0 '***' 0.001 '**' 0.01 '*' 0.05 '.' 0.1 ''						

Table S2. Output of PERMANOVA analysis for bacteria (A) and fungi (B) in the different biosystems during hemp retting process.

**A) Bacteria**		**Df**	**SumsOfSqs**	**R2**	**F**	**Pr(>F)**	
Permutation free	Field retting time	3	8.5656	0.40849	180.509	0.001	***
	Water retting time	4	6.4659	0.30836	102.195	0.001	***
	Field retting time : Water retting time	12	5.3045	0.25297	27.947	0.001	***
**B) Fungi**		**Df**	**SumsOfSqs**	**R2**	**F**	**Pr(>F)**	
Permutation free	Field retting time	3	6.3118	0.29842	75.993	0.001	***
	Water retting time	4	6.5080	0.30769	58.766	0.001	***
	Field retting time : Water retting time	12	7.2235	0.34152	21.742	0.001	***
Signif. codes: 0 ’***’ 0.001 ’**’ 0.01 ’*’ 0.05 ’.’ 0.1 ’ ’ 1							
